# Supplementary material for: The adequacy of tissue microarrays in the assessment of inter- and intra-tumoural heterogeneity of infiltrating lymphocyte burden in leiomyosarcoma
Source: Sci Rep. 2019 Oct 10;9:14602. doi: 10.1038/s41598-019-50888-5 (PMC6787212; doi:10.1038/s41598-019-50888-5)
Supplement: Supplementary file 1 — Supplemental info [file 41598_2019_50888_MOESM1_ESM.pdf]

Title: The adequacy of tissue microarrays in the assessment of inter- and intra-tumoural heterogeneity of infiltrating lymphocyte burden in leiomyosarcoma

Authors: Lee ATJ[1, 2], Chew W[2], Wilding CP[2], Guljar N[2], Smith MJ[1], Strauss DC[1], Fisher C[1], Hayes AJ[1], Judson I[1], Thway K[1], Jones RL[1,3,4] and Huang PH[2,4]

Affiliations: 1 Sarcoma Unit, The Royal Marsden NHS Foundation Trust, London SW3 6JJ, UK. 2 Division of Molecular Pathology and 3 Division of Clinical Studies, The Institute of Cancer Research, London, SW3 6JB, UK. 4These authors are joint senior authors.

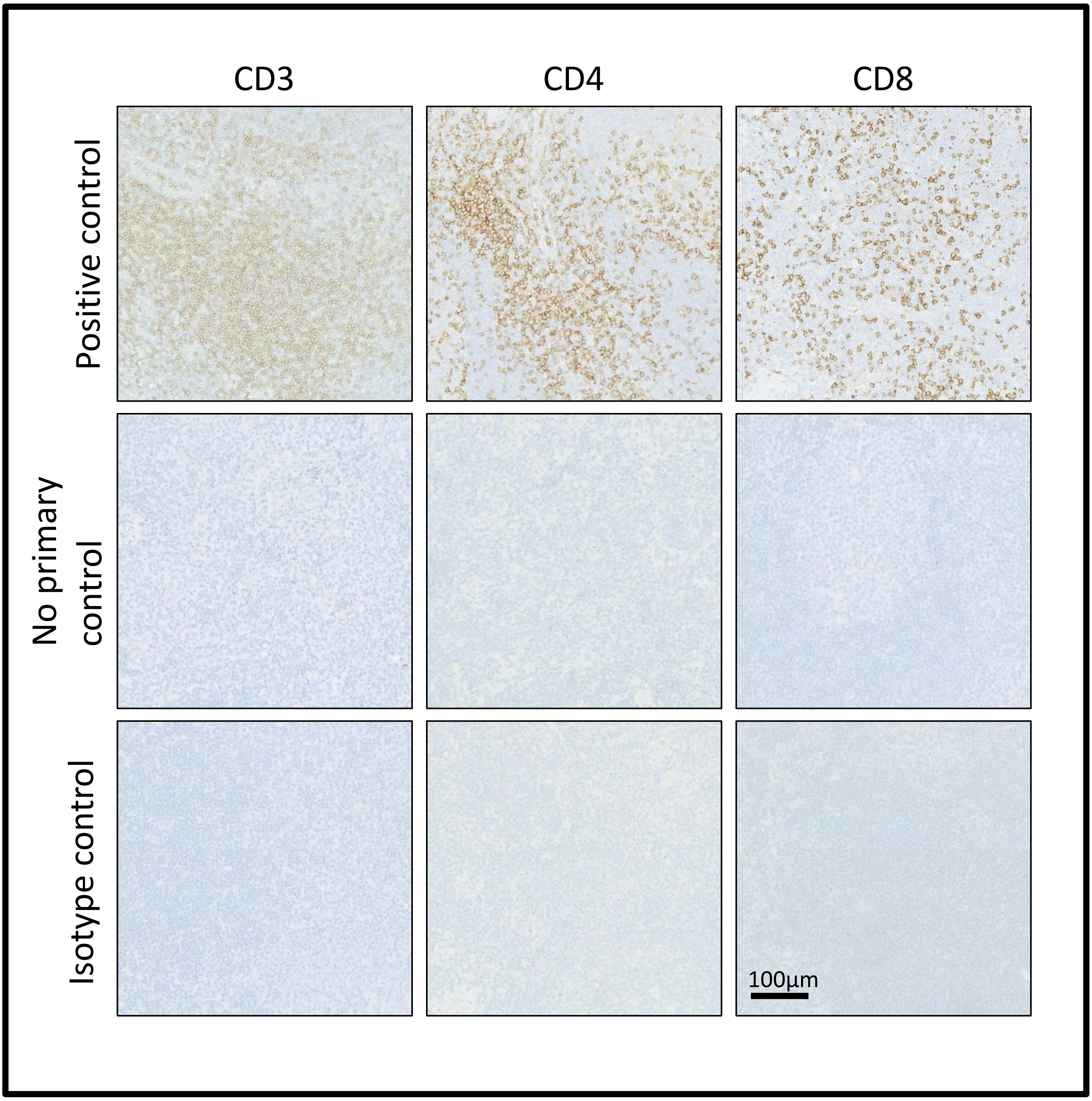

Figure S1

Figure S1: Immunohistochemical staining of human tonsillar control tissue

Representative IHC images at x20 magnification demonstrating positive, no primary and isotype control staining undertaken for CD3, CD4 and CD8.

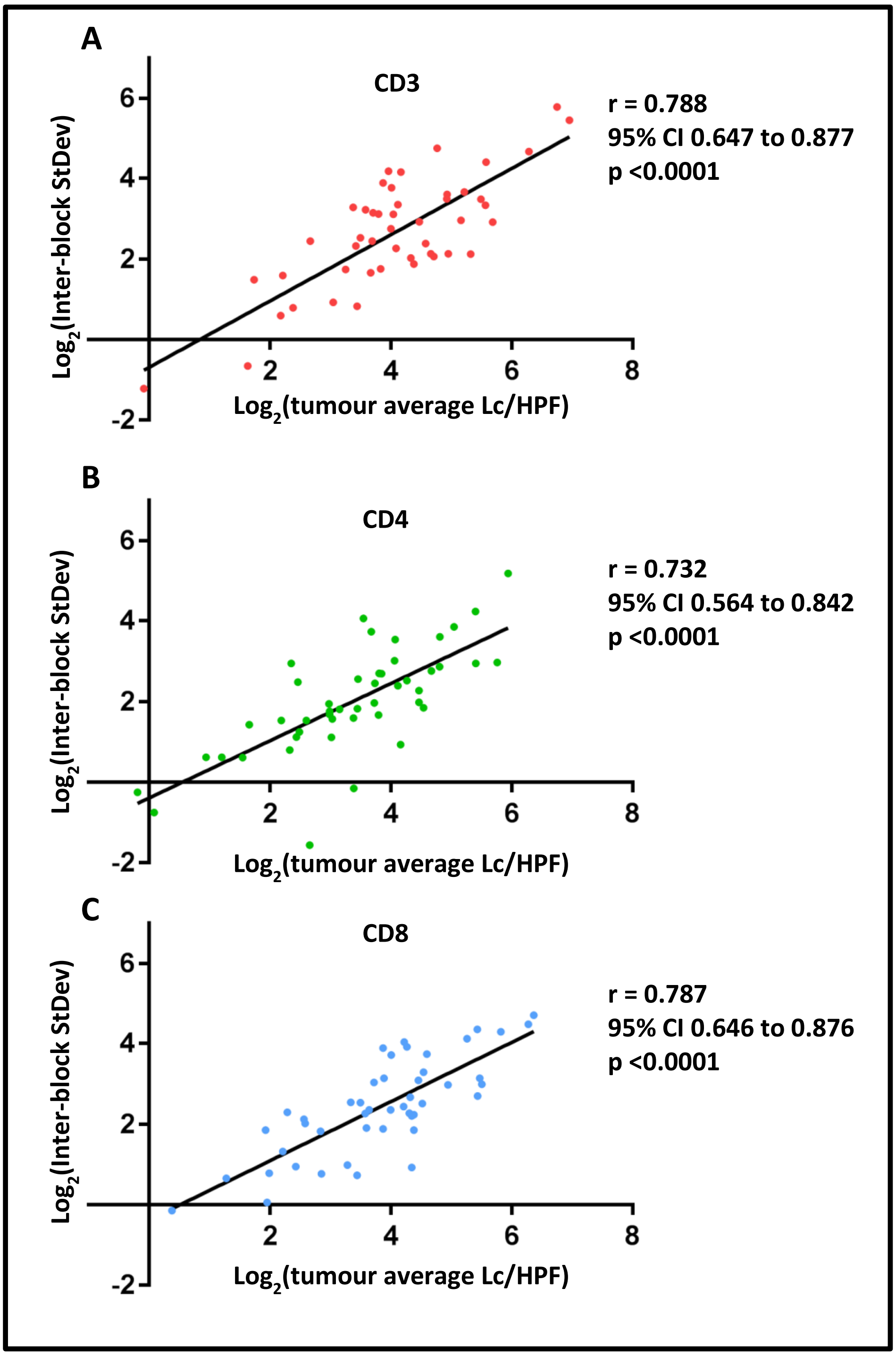

Figure S2

Figure S2: Assessment of the correlation between average lymphocyte count and inter-block standard deviation.

Dot plots demonstrate correlation between log_2-transformed values for tumour average TIL/HPF and the standard deviation of average TIL/HPF of constituent blocks for (A) CD3 (B) CD4 and (C) CD8. Pearson correlation coefficients (r) and associated 95% confidence intervals and significance levels are shown.

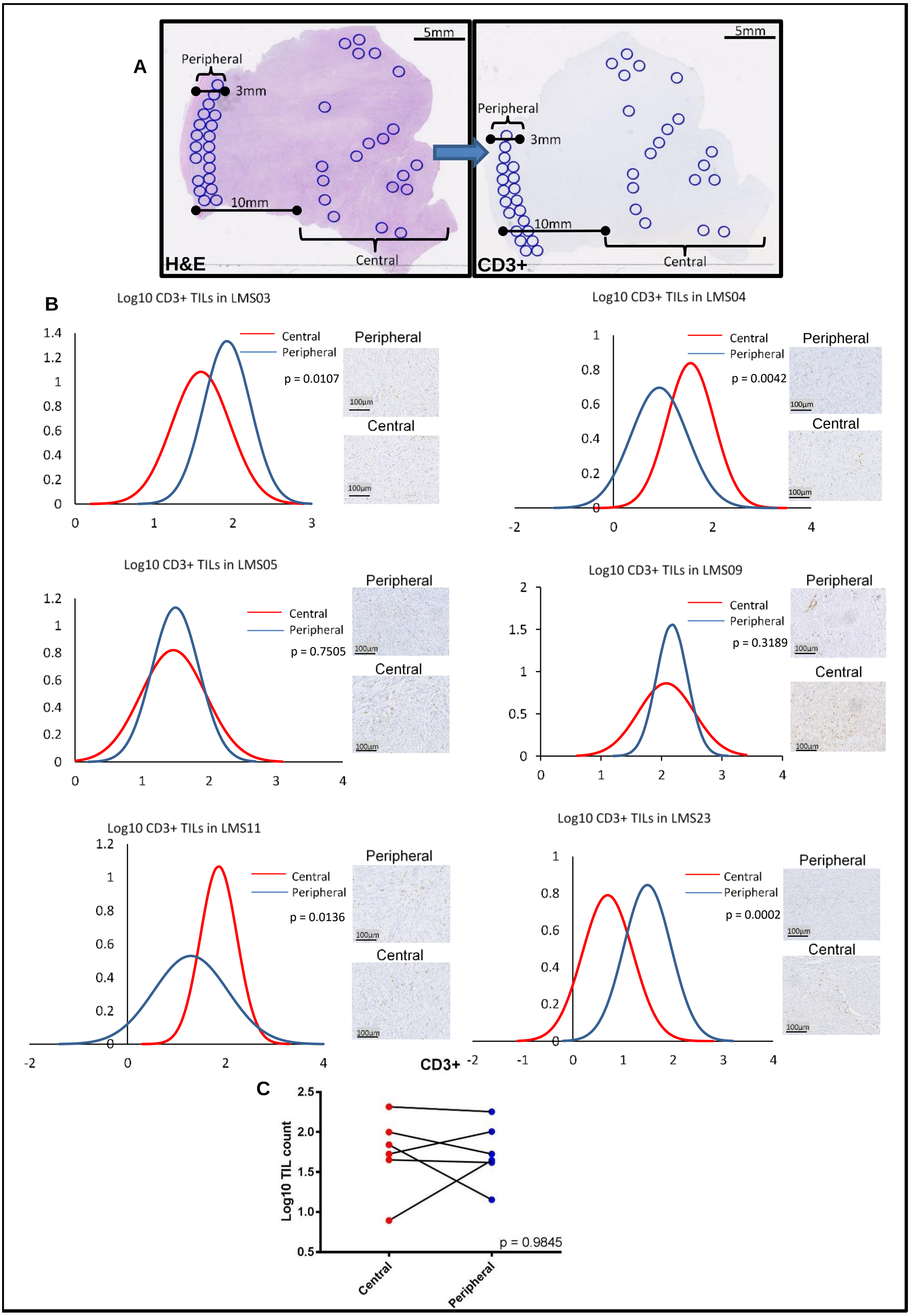
A
Peripheral
3mm
10mm
Central
5mm
H&E
CD3+
B
Log10 CD3+ TILs in LMS03
Central
Peripheral
p = 0.0107
Log10 CD3+ TILs in LMS04
p = 0.0042
Log10 CD3+ TILs in LMS05
p = 0.7505
Log10 CD3+ TILs in LMS09
p = 0.3189
Log10 CD3+ TILs in LMS11
p = 0.0136
Log10 CD3+ TILs in LMS23
p = 0.0002
100µm
C
Log10 TIL count
p = 0.9845
Central
Peripheral

Figure S3

Figure S3: Assessment of CD3+ TIL numbers in tumour central and peripheral regions.

A Overview diagram of the workflow of experimental approach. vTMA cores were placed within 3mm of an inked margin (tumour periphery) and vTMA cores were placed a minimum of 10mm from the inked margin (tumour centre). Each vTMA core was individually isolated as a digital image, and number of TILs determined through digital counting. **B** The CD3+ TIL counts for each virtual core were log10 transformed and used to determine the mean and standard deviation for the tumour centre and periphery of each LMS sample. These were then used to generate normal distribution curves for each of the 6 samples included in the centre vs periphery subgroup analysis, with an associated x20 magnification image of a representative portion of the vTMA core. The log10 TIL counts for the centre and periphery within each sample were then compared with each other using paired t-tests with p-values included. **C** Parallel dot plots of paired log10 TIL counts for central and peripheral regions for CD3+ lymphocytes . Paired t-test analysis revealed no significant difference in CD3+ TIL counts when assessing the 6 LMS cases together.

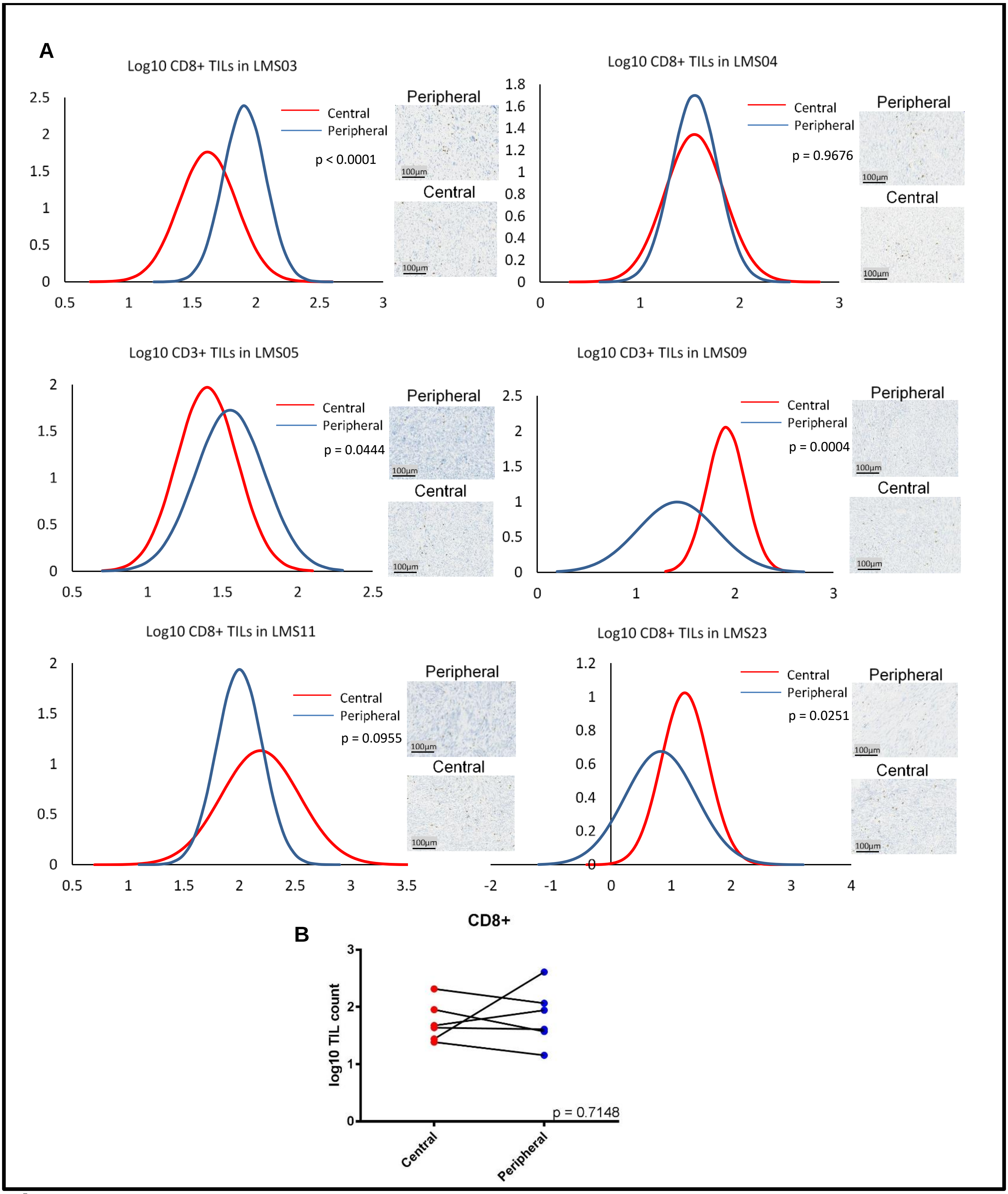
A
Log10 CD8+ TILs in LMS03
Central
Peripheral
p < 0.0001
Peripheral
Central
100µm
Log10 CD8+ TILs in LMS04
Central
Peripheral
p = 0.9676
Log10 CD3+ TILs in LMS05
Central
Peripheral
p = 0.0444
Log10 CD3+ TILs in LMS09
Central
Peripheral
p = 0.0004
Log10 CD8+ TILs in LMS11
Central
Peripheral
p = 0.0955
Log10 CD8+ TILs in LMS23
Central
Peripheral
p = 0.0251
B
CD8+
log10 TIL count
p = 0.7148
Central
Peripheral

Figure S4

Figure S4: Assessment of CD8+ TIL numbers in tumour central and peripheral regions.

A The CD8+ TIL counts of each virtual core were log10 transformed, and this data used to determine the mean and standard deviation for the centre and periphery of each of the 6 LMS samples included in this subgroup analysis. These were then used to generate normal distribution curves for each case, with an associated x20 magnification image of a representative portion of the vTMA core. Log10 TIL counts of the centre and periphery for each sample were then compared with each other using paired t-tests, with the resultant p value included on the normal distribution graphs. **B** Parallel dot plots of paired log10 transformed CD8+ TIL counts for central and peripheral regions across the 6 LMS samples. Paired t-test revealed no significant difference in CD8+ TIL count between the tumour centre and periphery when analysing all 6 LMS cases together.

Supplemental Methods

Immunohistochemistry

Immunohistochemistry was performed on consecutive 4-µm FFPE tumour sections cut afresh from each of the retrieved blocks for all included tumours. DAKO link automated stainer was used for all IHC processing. Tissue sections were deparaffinised with xylene then rehydrated with graded ethanol (100%, 95% to 80%).

Antigen retrieval was performed using DAKO FlexEnvision kit (K8002) with either pressure cooking at pH6 (CD3), incubation with PTM buffer at pH9 (CD4 and CD8)< or 18 minutes microwave heating at pH6 (CD20). All primary monoclonal antibodies were from DAKO as follows: CD3 (M0452, 1:600 dilution), CD4 (4B12, 1:80), CD8 (C8/144B, 1:100), CD20 (L26, 1:400). Slides were incubated with primary antibody for 60 minutes at room temperature and visualised using DAKO FlexEnvision (Rabbit/Mouse) kit (K8002), followed by application of DAB, resulting in visible brown colouration reaction at site of target antigen. Finally, nuclear counterstaining with haematoxylin was performed prior to coverslipping.

For positive controls, normal paraffin-embedded tissue from human tonsil, appendix and spleen was used. Omission of primary antibody during parallel staining of these tissues was used for negative controls.
